# Cracking-Resistance Mechanism of Fiber-Reinforced Coal-Based Solid-Waste Grouting Materials

**DOI:** 10.3390/ma19020389

**Published:** 2026-01-18

**Authors:** Shuai Guo, Weifeng Liang, Xiangru Wu, Chenyang Li, Hongzeng Li, Yahui Liu, Shenyang Ouyang, Yachao Guo, Junmeng Li

**Affiliations:** 1 School of Mines, China University of Mining & Technology, Xuzhou 221116, China; guoshuai@cumt.edu.cn (S.G.); wuxiangru0116@163.com (X.W.); ts24020140p31@cumt.edu.cn (C.L.); 15227713721@163.com (H.L.); ouyangshenyang@126.com (S.O.); yachaoguo2020@163.com (Y.G.); 2 Sandaogou Coal Mine, Yulin 719400, China; liangwf0031@163.com

**Keywords:** coal gangue, solid waste, fiber reinforcement, grouting material, performance analysis, cracking resistance, mechanism

## Abstract

Grouting technology can be employed to repair cracks in an aquifer to maintain its stability; however, existing grouting materials tend to come with problems such as low flexural strength, poor cracking resistance, and the coupled effects of fiber reinforcement and sulfoaluminate cement (SAC) addition on hydrate evolution, and pore-refinement and crack-resistance mechanisms in coal-based solid-waste cementitious grouts remain insufficiently understood. In this paper, fiber-modified coal-based solid-waste grouting (F-CWG) materials were prepared by mixing different contents of sulfoaluminate cement (SAC) and different fibers. The mechanical strength, microstructure, hydration products, and pore evolution characteristics were analyzed by means of mechanical property tests, energy-dispersive X-ray spectroscopy (SEM/EDS), X-ray diffraction (XRD), thermogravimetric analysis (TGA), and nuclear magnetic resonance (NMR). The results show that the mechanical strength decreases at first due to insufficient early-stage hydration products. Specifically, the 28 d compressive and flexural strengths decrease from 15.34 MPa and 4.55 MPa at 0% SAC to 8.18 MPa and 2.99 MPa at 40% SAC but increase again to 13.36 MPa and 3.79 MPa at 60% SAC as the formation of ettringite (AFt) and C–S–H is promoted with higher SAC content. Among the tested fibers, a dosage of 0.6% generally improves mechanical strength and refines pore structure, with PVA and steel fibers showing the most pronounced effects. Our results reveal the mechanism behind the enhancement of cracking resistance in F-CWG materials, providing a scientific basis for grouting and water-preservation mining, and are of great significance in improving the utilization rate of coal-based solid waste.

## 1. Introduction

Coal mining causes the development of overlying aquifer fractures and fissures, which leads to serious groundwater loss. Currently, grouting technology is predominantly employed to repair aquifer fractures and mitigate water loss [1,2]. However, existing grouting techniques usually use cement-based grouting materials [3], which are costly and less tough [4,5]. With the massive mining of coal resources, the cumulative accumulation of coal-based solid waste in China has been increasing year by year [6], and not only does the coal occupy a large amount of land space, but it also causes serious pollution to the environment [7,8]. There is an urgent need to dispose of and utilize coal gangue. The advantages of using coal-based solid waste as a raw material to prepare fissure grouting materials are the wide source of raw materials, the simple process, and low costs [9]. This method can effectively use up coal-based solid waste and has thus become a current research hotspot.

At present, research on the coal-based solid wastes used in water-plugging grouting and backfilling materials generally follows a “mix design–workability–mechanical performance” framework, with a particular emphasis on how the solid content, water-to-binder ratios/liquid-to-solid ratios, and fine-particle constituents affect slurry fluidity and strength. In cement-based or cement-like high-flow grout systems, researchers typically vary the solid mass fraction and water–cement ratio to clarify how fine particles regulate rheology and mechanical properties and have reported that increasing the moisture content leads to a reduction in the compressive strength of backfill bodies [10,11]. In alkali-activated/geopolymer systems, orthogonal experimental design, response surface methodology, and parameter optimization are commonly employed to quantify the relationships between initiator content, sodium silicate modulus, alkali solution proportions, and strength, generally exhibiting an “optimal range” or a “first increase then decrease” trend [12,13,14]. In addition, for multi-component solid-waste-based grouting materials, prior work has identified optimal combinations with both high compressive strength and improved impermeability by adjusting the coal gangue–fly ash–bentonite ratio and alkali content [15]. Other studies have introduced polymer modification or magnesium-based cementitious systems to enhance mechanical performance, refine microstructures, and explore mechanisms such as the influence of inorganic salt ions and CO_2_ sequestration via carbonation [16,17,18]. Meanwhile, the evolution of early-age pore structures and the process of interfacial reconstruction can markedly affect the subsequent mechanical performance and durability of these materials [19,20].

When a coal-based solid-waste grouting material is used to repair cracks in overlying rock, its flexural strength is low, and its cracking-resistance performance is poor [21,22]. At present, a common optimization method involves incorporating fibers into a coal-based solid-waste grouting material to enhance its mechanical properties [23]. Cao et al. [24] discussed the mechanical properties and damage evolution characteristics of waste tire steel fiber (WTSF)-modified backfill and revealed its influence mechanism in the structural weakening of backfill. Chinchillas et al. [25] incorporated polyvinylidene fluoride (PVDF) nanofibers into mortar mixtures and investigated the effects of different fiber dosages on the mechanical properties of the mortar. The results showed that the compressive strength and flexural strength increased by 12% and 23%, respectively, after 0.1% nanofibers were added. Zhang et al. [26] tested the properties of polyvinyl alcohol (PVA) fiber, nano-SiO_2_-modified fly ash, and metakaolin-based polymer/alkali-activated mortar, and the results showed that the addition of PVA fiber enhanced the compressive strength and fracture performance, and the optimal content was 0.8%~1.0%. Sahin et al. [27] studied the influence of different basalt fiber proportions on the mechanical properties of metakaolin base polymer mortar and found that when the basalt fiber was 0.8%~1.2%, the compressive strength and flexural strength of the polymer increased by 25% and 50%, respectively. Hwang et al. [28] added coconut fiber to a cement composite to study its mechanical properties, and the results showed that when the ratio of coconut fiber to mortar was 0%~4%, the 28 d flexural strength of the material increased from 5.2 MPa to 7.4 MPa.

Extensive research has also explored the utilization of coal-based solid wastes in foam concrete and alkali-activated cementitious materials. In foam concrete systems, the incorporation of coal dust can regulate pore structure and enhance compressive strength within an appropriate dosage range; however, excessive addition may increase water demand and destabilize the foam films, leading to pore coarsening and a decline in strength [29]. Meanwhile, studies based on a “mix design–foam stabilization–pore-structure evolution” perspective indicate that the synergistic use of coal gangue and other constituents can markedly influence the pore structure and strength of foam concrete. Further improvements can be achieved through particle-stabilization strategies; for example, using modified fly ash as an efficient solid-particle foam stabilizer can enhance foam stability and optimize the resulting pore structure and mechanical performance [30,31]. In geopolymer systems, fly ash serves as a primary precursor, and its intrinsic characteristics—such as amorphous phase content and particle size—significantly affect gel formation, porosity, and compressive strength; accordingly, the suitability of different fly ashes can be evaluated using comprehensive reactivity indices [32]. Moreover, incorporating other solid wastes such as copper slag can promote gel formation and further improve the strength of fly ash-based geopolymers [33].

The above studies show that fiber can effectively improve the mechanical strength and cracking-resistance of materials, but research on the cracking-resistance mechanism remains limited. To fill this knowledge gap, in this study, we develop fiber-modified coal-based solid-waste grouting materials and establish the relationships among mixture composition, microstructural evolution, and crack resistance, providing a basis for material design in grouting-based water-preservation mining and resource utilization in coal-based solid wastes. Unlike previous studies that primarily report performance improvements by focusing on a single factor—either solid-waste replacement or fiber addition—this work couples macroscopic mechanical behavior with the formation of hydration products and pore structure evolution, thereby elucidating the synergistic regulation of SAC dosage and fiber reinforcement and its influence on crack-resistance mechanisms. Consequently, the present study not only provides mechanistic evidence for crack-resistance enhancement in coal-based solid-waste grouting materials but also offers practical guidance for mixture optimization and material selection in engineering applications.

## 2. Objectives and Scope

In this study, we aimed to develop coal-based solid-waste grouting mortars with improved crack resistance through the coupled design of sulfoaluminate cement (SAC) content and fiber reinforcement and to clarify the corresponding material–structure–property relationships. Coal gangue and fly ash were used as the primary solid-waste constituents, and four types of fibers (steel fiber, glass fiber, polyvinyl alcohol fiber, and basalt fiber) were incorporated as reinforcements.

In this work, we take coal-based solid waste such as coal gangue and fly ash as raw materials and blend basalt fiber (BF), glass fiber (GF), polyvinyl alcohol fiber (PVA), and steel fiber (SF) to prepare fiber-modified coal-based solid-waste grouting (F-CWG) materials. By means of mechanical property tests, SEM/EDS, XRD, thermogravimetric analysis (TGA), and nuclear magnetic resonance (NMR), we analyze the mechanical strength, microstructure, hydration products, and pore structure characteristics of materials under different cement composite ratios and fiber types to reveal the enhancement mechanism of the fibers with regard to the mechanical strength and cracking-resistance properties of materials. This study provides a scientific basis for grouting-based water-preservation mining and coal-based solid-waste disposal, which is of great significance in reducing environmental pollution.

## 3. Materials and Methods

### 3.1. Raw Materials and Mix Ratio Design

#### 3.1.1. Raw Materials

**Figure 1:**
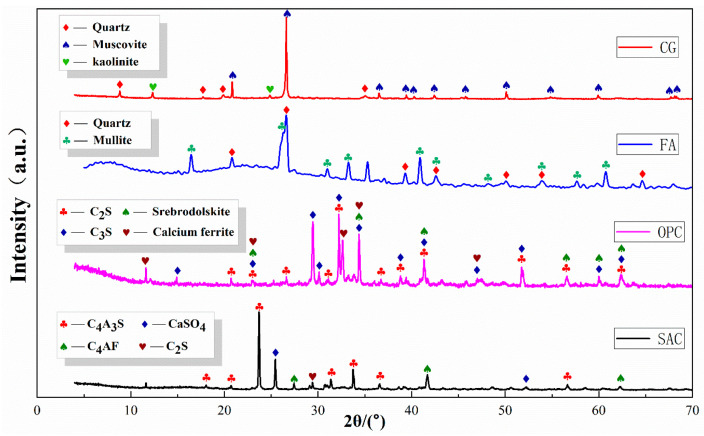
XRD patterns of raw materials.

**Figure 2:**
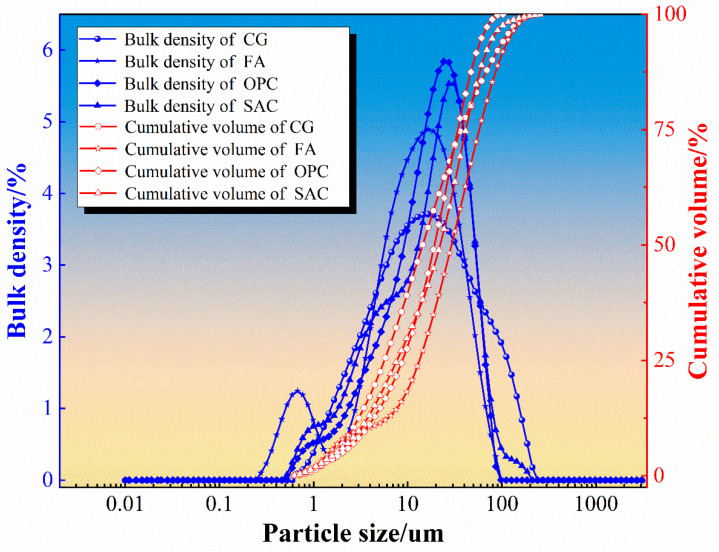
Particle size distribution of raw materials.

The coal gangue (CG) used in this study was taken from the Yuanyanghu mining area in Ningxia. After the coal gangue was crushed and ground with a crusher and ball mill, gangue powder was obtained after passing it through a 325-mesh square screen [34,35]. Fly ash (FA) was obtained from a power plant at the Ningdong Energy and Chemical Industry (Yinchuan, Ningxia, China) and the main particle size distribution was 0.1~100 um. Ordinary Portland cement (OPC) and sulfoaluminate cement (SAC) were bought from Zhengzhou Jinghua Special Cement Co., Ltd. (Zhengzhou, China). The XRD patterns and particle size distributions of the four raw materials are shown in Figure 1 and Figure 2.

The main mineral phases of CG are quartz, muscovite, and kaolinite, which are hard in texture and have good bearing capacity. The main mineral phases of FA are quartz and mullite, which have a large specific surface area and strong water absorption. The main mineral phases of OPC are C_2_S, srebrodolskite, C_3_S, and calcium ferrite, which are high-strength and fast-setting. The SAC used in this study is a sulfoaluminate–belite-type cement. It is mainly composed of ye’elimite (C_4_A_3_Ŝ) and anhydrite (CaSO_4_), with belite (β-C_2_S) present. A minor ferrite phase (C_4_AF) is also detected. The XRF oxide composition of this cement is provided in Table 1.

In Figure 2, the blue curves represent the differential particle size distribution (distribution density), indicating the proportion of particles within each size interval, while the red dashed curves show the cumulative volume distribution, i.e., the cumulative percentage of particles smaller than a given size. FA exhibits a D_50_ of 13.38 μm and a D_90_ of 42.19 μm, indicating the highest fine-particle fraction and the greatest fineness among the tested materials. In contrast, OPC and SAC have D_50_ values of 19.13 μm and 19.28 μm, respectively, suggesting slightly coarser and relatively more concentrated size distributions. CG shows a D_50_ of 16.40 μm but a much larger D_90_ of 85.84 μm, implying the broadest particle-size distribution and a pronounced coarse tail. Therefore, FA is more favorable in particle packing and matrix densification, whereas the wide gradation and coarse tail of CG can provide a skeletal/grading effect within the composite system.

The water-reducing agent is a polycarboxylic acid-based high-performance water-reducer (synthesized from polyether monomers such as TPEG/HPEG, acrylic acid, maleic anhydride, etc., with an average molecular weight ~8000) produced by Jiangsu Subote New Materials Co., Ltd. (Changzhou, Nanjing, China). The parameters are shown in Table 2.

**Figure 3:**
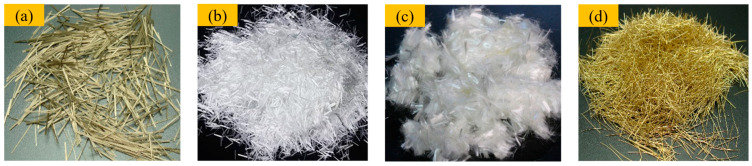
Different fiber types: (**a**) BF, (**b**) GF, (**c**) PVA, and (**d**) SF.

In order to improve the performance of materials, four types of fibers, basalt fiber (BF), glass fiber (GF), polyvinyl alcohol fiber (PVA), and steel fiber (SF), were selected for the preparation of F-CWG materials. The appearance and performance parameters of fibers are shown in Figure 3 and Table 3.

BF and SF appear yellow, whereas GF and PVA are white. All four fibers were cut to a length of 10 mm, which facilitates crack bridging in the cementitious matrix. In terms of density, SF has the highest density (7.80 g/cm^3^), BF and GF are intermediate (3.05 and 2.59 g/cm^3^, respectively), and PVA has the lowest density (1.31 g/cm^3^). BF, GF, and PVA have similar diameters of approximately 15 μm and are therefore classified as fine fibers; at the same volume fraction, their higher number density allows more fibers to participate around microcracks, and this result is beneficial in enhancing crack resistance and toughness. In contrast, SF has a much larger diameter (0.30 mm) and is a coarse fiber, providing higher load-carrying capacity per filament and proving more effective in bridging wider cracks. Regarding elongation at break, BF and GF exhibit lower values, whereas PVA and SF show higher elongation, indicating better ductility and a greater potential to improve the toughness and crack resistance of the composite.

#### 3.1.2. Test Proportioning Design

In order to improve the performance of F-CWG materials, the material ratio should be determined according to the pre-test. In this study, the optimal ratio was CG–FA–cement = 5:1:4, the water–cement ratio was 0.4, and water-reducing agent content was 1.0%. Based on the pre-experimental results, the cement composite system was formed by adding SAC into OPC at a proportion of 10%, step by step [36,37], and the material ratio scheme is shown in Table 4. The mechanical strength and cracking resistance of F-CWG materials incorporating BF, GF, PVA, and SF at fiber volume fractions of 0.2%, 0.4%, 0.6%, 0.8%, and 1.0% were investigated, with the cement composite ratio and fiber type serving as variables.

### 3.2. Sample Preparation and Curing

**Figure 4:**
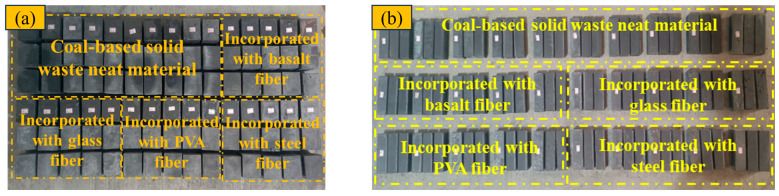
Preparation of material specimens: (**a**) compressive specimens and (**b**) flexural specimens.

Coat the inner wall of the mortar test mold evenly with the release agent. Weigh a certain quality of CG, FA, OPC, SAC, and other raw materials into the mixing bucket, and stir for 3 min. Then, add the fiber and stir again for 3 min, and, finally, add a certain quality of water to stir and mix for 3 min. After the slurry is evenly mixed, pour it into the test molds of 70.7 mm × 70.7 mm × 70.7 mm and 40 mm × 40 mm × 160 mm, respectively, and use a scraper to smooth the upper surface. After 24 h of casting, demold it and place it in a room-temperature environment with water spray for 28 d. The compressive and flexural specimens obtained are shown in Figure 4. All experimental programs were tested using three parallel specimens to minimize random error and improve result reliability.

### 3.3. Experimental Methods

**Figure 5:**
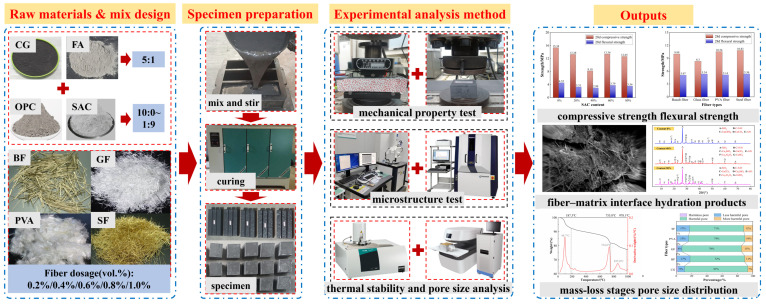
The specific preparation process of F-CWG materials.

The preparation steps for F-CWG materials are shown in Figure 5. We tested the macroscopic mechanical properties of materials, such as compressive strength and flexural strength, and analyzed the microstructure of materials using SEM-EDS, XRD, TGA, and NMR.

#### 3.3.1. Mechanical Property Test

According to the Standard for Test Methods of Concrete Physical and Mechanical Properties (GB/T 50081-2019), the mechanical properties of materials were tested using the WDW-300 electro-hydraulic servo testing machine (Jinan Times Test Machine Co., Ltd., Jinan, China). The test system utilized displacement-controlled loading at a loading rate of 2 mm/min and a fracture elongation of 70% of the peak load [38]. The stress–strain curves were obtained when the loading was stopped, and the compressive strength and flexural strength of the specimens were calculated separately.

#### 3.3.2. SEM/EDS Test

After the specimens cured for 28 d were dried, thin-slice samples of about 2 cm × 2 cm × 0.5 cm were obtained from specimens. The microstructure of materials was analyzed with a Zeiss Sigma 300 scanning electron microscope (Zeiss, Oberkochen, Germany) and Oxford Xplore 50 energy spectrometer (Oxford Instruments, Abingdon, United Kingdom). During the experiment, the specimens were fixed with conductive adhesive and sprayed with gold to improve conductivity [15].

#### 3.3.3. XRD Test

The mineral composition of materials was analyzed with a Rigaku Ultima IV X-ray diffractometer (Rigaku Corporation, Tokyo, Japan). Samples were dried, ground, placed centrally in the groove, lightly pressed with a glass slide to remove excess, and tested. The scanning range was 5°~75° (2θ), and the scanning speed was set to 5°/min.

#### 3.3.4. TGA Test

The effects of different cement composite ratios and types of fibers on the hydration products of the materials were tested using a TG 209 F1 Libra thermogravimetric analyzer (Netzsch, Selb, Germany). After drying, the samples were preliminarily ground and then subjected to high-speed grinding in a grinder, packed into bags, and placed in thermogravimetric crucibles to record mass changes. The testing parameters were set as follows: heating range, 0–800 °C; and heating rate, 10 °C/min.

#### 3.3.5. NMR Test

The MacroMR12-150H-I nuclear magnetic resonance system was used to test the effect of the cement composite ratio and fiber type on the pore structure of materials. The magnet type of the test system is a permanent magnet with a strength range of 0.3 ± 0.05 T, a resonance frequency of 12 MHz, and a probe coil diameter of 110 mm. Cube samples with dimensions of 50 mm × 50 mm × 50 mm were used for the test.

## 4. Results and Discussion

### 4.1. Mechanical Strength Evolution Laws of Materials

**Figure 6:**
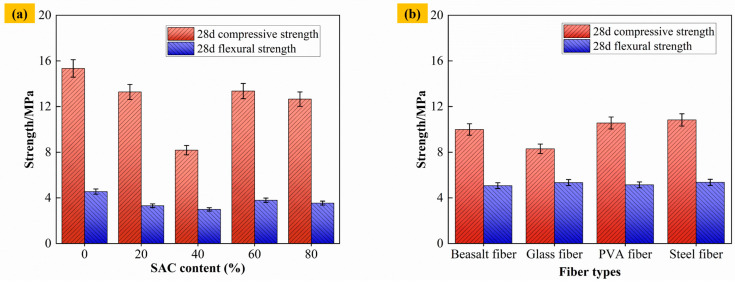
Compressive and flexural strength of materials: (**a**) different contents of SAC and (**b**) different fiber types.

Figure 6a shows the compressive strength and flexural strength of materials under different cement composite ratios. When the content of SAC is 0%, the maximum compressive strength of materials is 15.34 MPa. When the content of SAC increases to 20% and 40%, the compressive strength of materials decreases significantly. This is because the hydration of SAC may increase the number of microcracks within the composite cement system, leading to a decrease in the overall strength [39,40]. When the content of SAC continues to increase to 60% and 80%, a large amount of anhydrous calcium thioaluminate in SAC reacts to form ettringite crystals, which strengthen the three-dimensional skeleton structure formed by gangue aggregate, fly ash particles, and C-S-H gel and further increase the strength of materials. The flexural strength of materials fluctuates with the increase in the content of SAC, showing the trend of “first decreasing and then increasing”, but the overall change is relatively small, indicating that the effect of SAC content on the flexural strength of materials is relatively limited.

Figure 6b shows the compressive and flexural strengths of materials of different fiber types at 0.6% content. Taking 40% SAC content in Figure 6a as the control group, the compressive and flexural strength of materials increases after the addition of BF, GF, PVA, and SF, but the increase in the compressive strength of F-CWG materials doped with GF is small. This is because, during the preparation of F-CWG materials, the incorporation of GF tends to increase slurry viscosity, which may lead to higher entrapped air and internal porosity, as well as less uniform fiber dispersion. As a result, the strengthening effect of GF is limited and the strength gain may be reduced compared with other fiber types [41,42].

### 4.2. Microstructure Analysis of Materials

#### 4.2.1. Microstructure Characteristics of Materials Under Different Cement Composite Ratios

**Figure 7:**
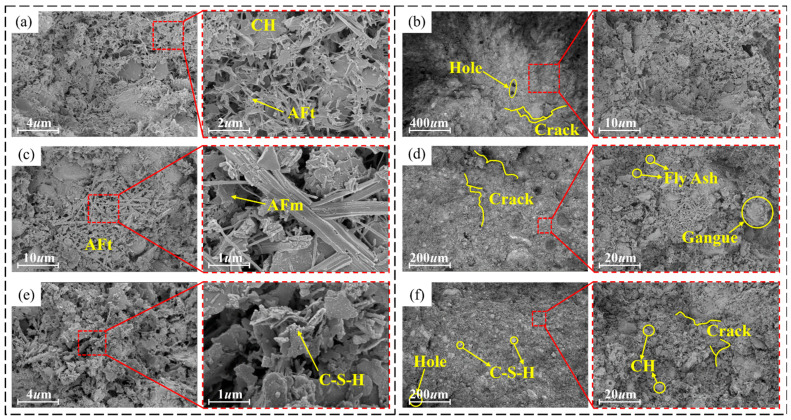
Microstructure of materials with different contents of SAC: (**a**,**b**) 0%, (**c**,**d**) 40%, and (**e**,**f**) 90%.

Figure 7a–f show SEM images of materials with SAC contents of 0%, 40%, and 90%. When the SAC content is 0%, Ca(OH)_2_ and AFt are formed rapidly and closely in the initial stage of the reaction, and there are fewer fly ash particles on the surface of the material. The surface structure of the material is more compact with smaller pore size and lower distribution density at this content compared to other contents [43]. When the content of SAC is 40%, the surface of the material is formed by AFm, which forms a three-dimensional network structure with AFt, C-S-H, gangue powder, and fly ash particles. When the content of SAC is 90%, a large number of hydration gel products, such as AFm, AFt, C-S-H, and CH, are generated on the surface of the material. Compared with the material with 40% SAC content, the surface of the material with this content formed a relatively dense three-dimensional structure due to a large number of hydration products attached to the CH surface.

**Figure 8:**
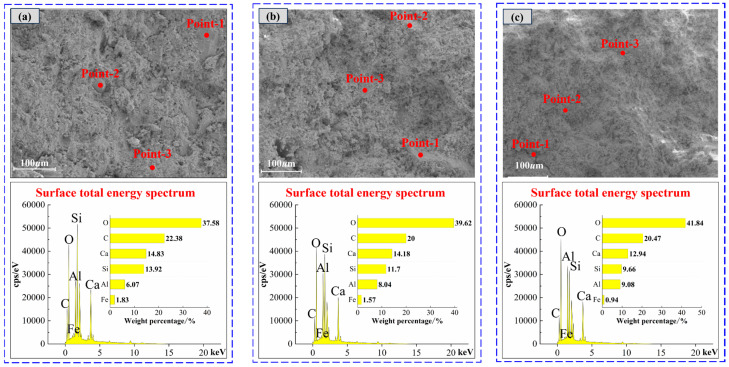
Energy spectrum analysis of clean pulp materials with different SAC contents: (**a**) 0%, (**b**) 40%, and (**c**) 90%.

Figure 8 shows the total energy spectrum of EDS surface of materials with different SAC contents. With the increase in SAC content from 0% to 90%, the elements’ weight percentages change: O increases, C and Al are relatively stable, and Si decreases. Combined with Table 5, it can be seen that the surface total energy spectrum of materials is consistent with the weight percentage of elements at different selected points, all of which contain four elements: O, C, Al, and Si. After the hydration of the cement and the activation of the fly ash, C-A-S-H, C-S-H, needle-rod AFt, and flake CH are mainly formed on the surface of the material. When the SAC content increases, the percentage of Al increases. This is because Al may be incorporated into the C–S–H structure, potentially forming C–A–S–H, thereby affecting the microstructure of the material [44].

#### 4.2.2. Microstructure Characteristics of Materials Under Different Fiber Types

**Figure 9:**
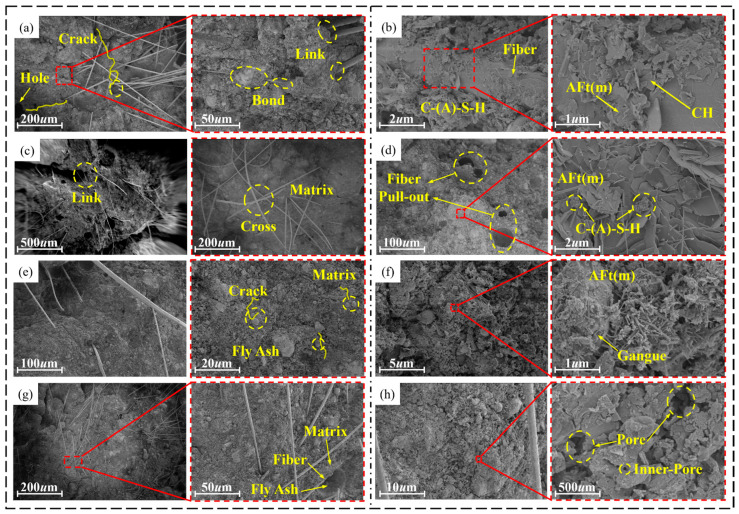
Microstructure of F-CWG materials with different fibers: (**a**,**b**) BF, (**c**,**d**) GF, (**e**,**f**) PVA, and (**g**,**h**) SF.

Figure 9a–g are SEM images of F-CWG materials under different fiber types. There are three types of links between fiber and matrix. Crack bridging: As shown in Figure 9a,c, the fibers act as a “bridge”, connecting substrates that are far apart to prevent the spread of cracks, thereby enhancing the cracking resistance of materials [45]. Matrix anchoring: As shown in Figure 9b, when the fiber is completely buried in the matrix, although the cracking resistance is reduced, it still provides a better bonding effect than when it is partially buried in the matrix [46,47]. Fiber interlocking: As shown in Figure 9c,d, the fibers are interlaced with the matrix to form a monolithic structure, which enhances the cracking resistance of the material. As shown in Figure 9e–h, the fibers are linked to the matrix in three ways: link, bond, and cross. By forming a dense three-dimensional network structure with gangue powder, fly ash particles, and other products, crack development is prevented.

**Figure 10:**
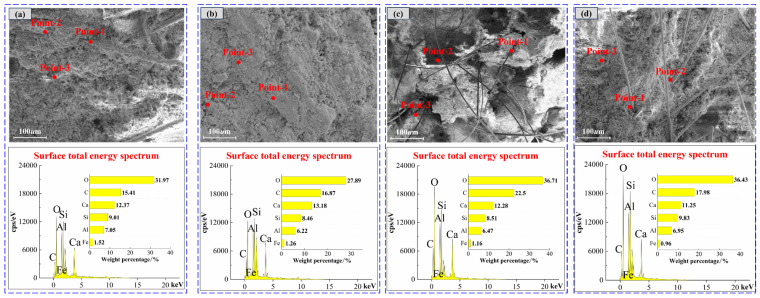
EDS spectra of different fibers after doping: (**a**) BF, (**b**) GF, (**c**) PVA, and (**d**) SF.

EDS spectrum analysis is performed on F-CWG materials mixed with different fiber types, and the results are shown in Figure 10 and Table 6. The distribution and percentage of Ca, Al, Si, and O are consistent with the 40% SAC content material. It is speculated that the fibers cross the surface of F-CWG materials, resulting in a decrease in the proportion of elements in the corresponding region. Note that the EDS analysis results represent the semi-quantitative elemental composition of the sample surface.

### 4.3. Analysis of Evolution Characteristics of Material Hydration Products

In most cases, fiber does not directly participate in the chemical reaction inside the material [48,49], so this section only reports XRD tests on the mineral phase structure of materials with different cement composite ratios.

#### 4.3.1. Evolution Characteristics of Hydration Products of Different Cement Composite Ratios

**Figure 11:**
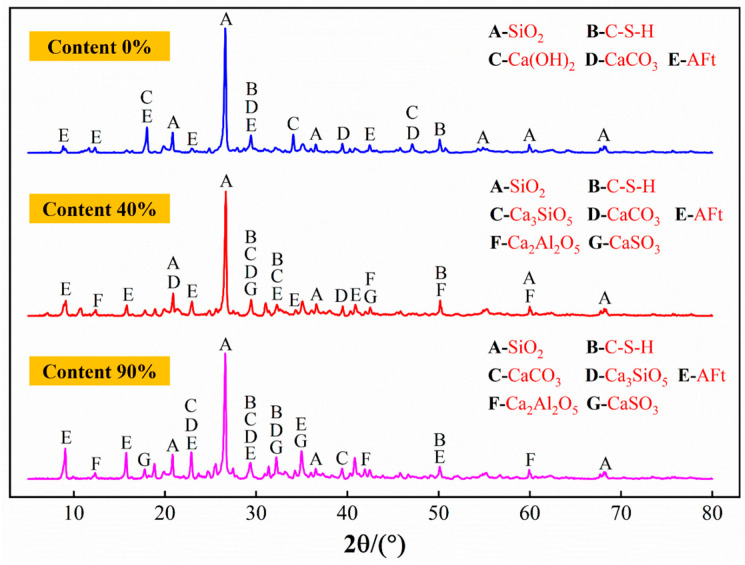
XRD patterns of materials with different cement composite ratios.

The XRD patterns of materials with different SAC contents are shown in Figure 11. When the SAC content is 0%, the mineral phases are mainly SiO_2_, C-S-H, Ca(OH)_2_, and CaCO_3_. When the SAC content increases to 40%, diffraction peaks of Ca_2_Al_2_O_5_ and CaSO_4_ appear in the pattern. The diffraction peaks of C-S-H and AFt decrease, indicating that the generated C-S-H is further consumed by the hydration reaction [50]. When the SAC content is 90%, Ca_2_Al_2_O_5_, CaSO_4_, and AFt diffraction peaks are increased, which can indicate that the high content of SAC promotes the generation of Ca_2_Al_2_O_5_, CaSO_4_, and AFt, contributing to the improvement of the material’s strength and reduction of its porosity. However, when there is too much SAC, the initial strength may be improved, but the strength may grow slowly in later stages, which will affect the overall performance of the grouting material.

**Figure 12:**
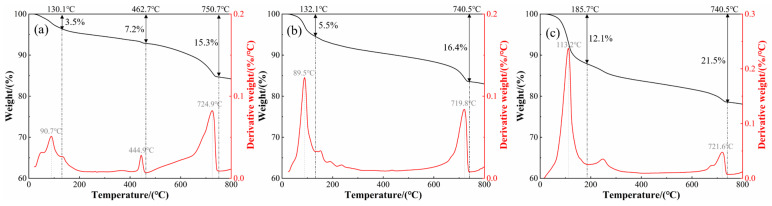
TG-DTG curve of coal-based solid-waste pulp: (**a**) content 0%, (**b**) content 40%, and (**c**) content 90%.

The TG-DTG curves of materials with different SAC contents are shown in Figure 12. The thermal decomposition characteristics of materials change with increasing SAC content. When the SAC content is 0%, heat-absorption peaks appear at 90.7 °C, 444.9 °C, and 724.9 °C, corresponding to the decomposition evaporation of AFt, C-S-H, and free water in pores and the decomposition of CH and CaCO_3_ [51,52]. With 40% SAC content, the peak position of heat absorption changes, the thermal stability of the material decreases, and the decomposition degree of CaCO_3_ intensifies. When the content of SAC increases to 90%, the mass loss ratio of each stage increases significantly, and in the middle and high-temperature stage, carbonate, silicate, and other minerals decompose violently, and the thermal stability decreases significantly [53]. In summary, the content of SAC will significantly affect the mineral decomposition behavior of the material, and the position of the heat-absorption peak will change with the increase in SAC content.

#### 4.3.2. Evolution Characteristics of Hydration Products of Different Fiber Types

**Figure 13:**
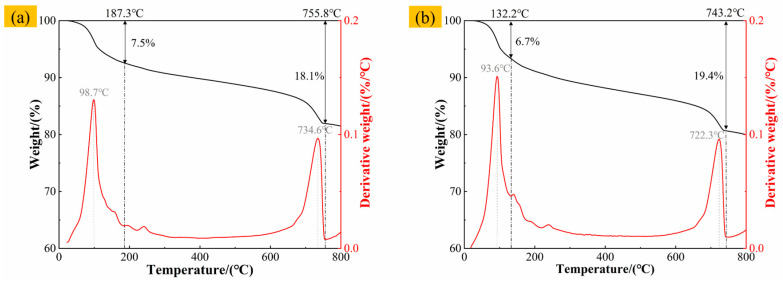

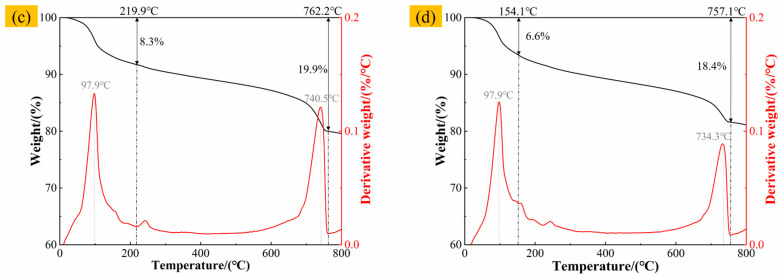
TG-DTG curves of different F-CWG materials after adding 0.6% fiber: (**a**) BF, (**b**) GF, (**c**) PVA, and (**d**) SF.

The TG-DTG curves of F-CWG materials with different fiber types at 0.6% content are shown in Figure 13. All four specimens exhibit two main mass-loss stages within 0–800 °C. The first stage occurs below approximately 200 °C, where a pronounced DTG peak is observed for all fiber-reinforced mixes, with peak temperatures of 98.7 °C, 93.6 °C, 97.9 °C, and 97.9 °C, respectively, mainly attributable to the evaporation of free and adsorbed water. The corresponding mass losses are 7.5%, 6.7%, 8.3%, and 6.6%, with the PVA-containing system showing the most pronounced loss in this stage. The second stage is concentrated at approximately 600–800 °C, where clear DTG peaks appear within 722.3–740.5 °C; the mass losses in this range are 18.1%, 19.4%, 19.9%, and 18.4%, which are associated with the decomposition of hydration products and related constituents. When heated to 800 °C, the total mass losses of the specimens are 18.52%, 20.03%, 20.44%, and 19.89%, respectively. Overall, the PVA system shows the poorest thermal stability, whereas the BF- and SF-reinforced systems exhibit smaller cumulative mass losses and thus better thermal stability, with the GF system falling in between.

### 4.4. Analysis of Materials’ Pore Evolution Characteristics

#### 4.4.1. Pore Size Variation

**Figure 14:**
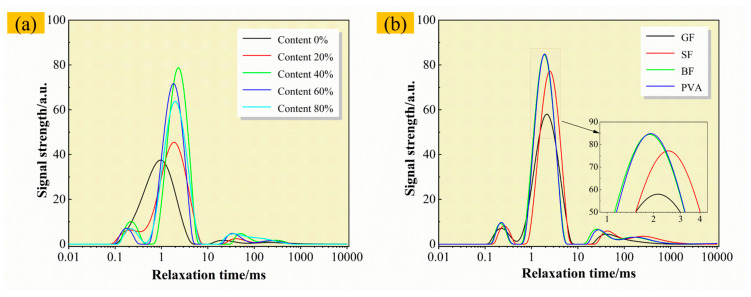
T_2_ spectra of materials: (**a**) different cement composite ratios and (**b**) different fiber types.

The T_2_ spectra of materials with different cement composite ratios are shown in Figure 14a. When the SAC content is 0%, the spectrum shows a “single-peak” structure and the position is left. When the content of SAC is different, the spectrum of the material presents a “triple-peak” structure [54]. Moreover, with the increase in the cement composite ratio, the peak value shifts to the left relaxation time, indicating that the average pore size decreases and the material density increases [55]; that is, a denser structure will be produced with a high content of SAC. The T_2_ spectra of F-CWG materials mixed with different fiber types are shown in Figure 14b. In this case, a “triple-peak” structure also appears, and the peaks of BF and GF may appear at lower relaxation times than those of PVA and SF.

#### 4.4.2. Aperture-Type Characteristics

Based on previous studies, the pore size–porosity distribution curves of materials can be classified into four categories: harmless pores (d < 20 nm), less harmful pores (20–50 nm), harmful pores (50–200 nm), and severely harmful pores (d > 200 nm) [48].

**Figure 15:**
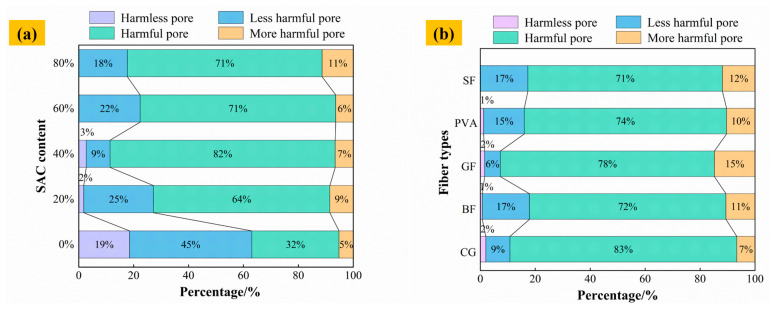
The proportion of material pore size type: (**a**) different cement composite ratios and (**b**) different fiber types.

The pore size types of materials with different cement composite ratios are shown in Figure 15a. When the SAC content is 0%, the proportion of harmful pores and more harmful pores is about 37%. The percentage of harmful pores and more harmful pores increases to 73%, 89%, 77%, and 82% when the SAC content is 20%, 40%, 60%, and 80%, respectively. When the content of SAC exceeds 60%, the harmless holes in the material almost disappear. This is because after the incorporation of SAC, the hydration products generated, such as AFm, AFt, and C-S-H, fill the pores, resulting in the failure of the small pore structure [56].

The pore size types of materials with different fiber types are shown in Figure 15b. In the control group with 40% SAC content, the proportion of multiple holes is 82%. After adding BF, GF, PVA, and SF, the proportion of multiple-damage pores in different F-CWG materials decreases by 10%, 4%, 8%, and 11%, respectively, compared with the control group; that is, the proportion of multiple-damage pores reduces after adding fibers. This is because the incorporation of fibers changes the pore structure of the material and forms a stable three-dimensional network structure, which contributes to the formation of smaller, less harmful pores and a denser structure [57].

## 5. Conclusions and Future Outlook

### 5.1. Conclusions

In this study, mechanical-strength tests, SEM/EDS, XRD, TGA, and NMR were used to analyze the mechanical properties, microstructure, hydration products, and pore evolution of materials under different cement composite ratios and different fiber types and to reveal the mechanism behind fiber enhancement improving the mechanical strength and crack resistance of materials. Our specific conclusions are as follows:

1. As the SAC content increased from 0% to 40%, the 28 d compressive and flexural strengths decreased from 15.34 MPa and 4.55 MPa to 8.18 MPa and 2.99 MPa, respectively, and then recovered as the SAC content increased further. In the fiber-modified system, taking the 40% SAC mixture as the reference, fiber incorporation enhanced both compressive and flexural strengths, and within the investigated range, a fiber volume fraction of 0.6% provided the best overall performance.
2. Different SAC contents will change the type and distribution of hydration products on the surface of materials, resulting in a C-A-S-H, which forms a dense three-dimensional structure and affects the mechanical strength of the material.
3. Higher SAC content promotes the generation of hydration products such as Ca_2_Al_2_O_5_, CaSO_4_, and AFt and improves the pre-strength of the material. The materials doped with different fibers all experience three stages—water evaporation, mineral decomposition, and the destruction of inorganic components—after being heated. When the temperature rises to approximately 800 °C, the mass loss of F-CWG materials incorporating BF, GF, PVA, and SF is 18.52%, 20.03%, 20.44%, and 19.89%, respectively. Therefore, in terms of thermal stability, BF and SF perform the best, followed by GF, and PVA performs the worst.
4. The T_2_ spectra of the materials with different cement composite ratios and different fiber types exhibit a “triple-peak” structure, and the SAC, which is present in large amounts, fills the pores by generating hydration products to form a denser structure. Fiber doping can build a stable three-dimensional network structure, reduce the proportion of multi-hazardous pores (d ≥ 200 nm), and improve the overall performance of the material.

### 5.2. Future Outlook

This research provides a foundation for the application of F-CWG materials in field grouting repair; however, studies on the long-term evolution mechanisms behind mechanical damage in these materials remain insufficient. In particular, the long-term mechanical degradation mechanism of fiber in F-CWG materials in complex multi-scene environments still needs to be further studied.

From a materials science perspective, future work will establish quantitative relationships among hydration evolution, pore structure changes, and fiber–matrix interfacial interactions to clarify their effects on crack resistance and durability. From an engineering perspective, the proposed coal-based solid-waste fiber-reinforced grouting mortar can be applied to sealing aquifer fractures and supporting water-preservation mining, and it is also suitable for leakage control and repair grouting in underground construction and infrastructure maintenance. The performance metrics and mix design recommendations reported in this study can support on-site material selection and quality control and may provide references for developing technical guidelines and engineering decision-making. In future studies, we will also systematically document macroscopic fracture characteristics of failed specimens and, together with SEM-based interfacial observations, conduct multi-scale failure analysis to obtain more direct evidence for the crack-resistance mechanisms.

## Figures and Tables

**Table 1:** The chemical composition of SAC (% by weight).

Raw Materials	Loss	SiO_2_	CaO_2_	Al_2_O_3_	Fe_2_O_3_	MgO	SO_3_
SAC	0.41	7.61	42.23	32.87	1.78	1.75	10.43

**Table 2:** Technical parameters of water-reducing agent.

Recommended Dosage	Water-Reducing Rate	Gas Content	Bleeding Rate	pH
0.1~1.5%	45%	2.5%	30%	7.0

**Table 3:** Fiber performance parameters.

Materials	Density	Length	Elongation at Break	Diameter
**BF**	3.05 g/cm^3^	10.00 mm	≤3.5%	15.20 μm
**GF**	2.59 g/cm^3^	10.00 mm	≥2.5%	14.80 μm
**PVA**	1.31 g/cm^3^	10.00 mm	≤30%	15.30 μm
**SF**	7.80 g/cm^3^	10.00 mm	≥25%	0.30 mm

**Table 4:** Coal-based solid-waste grouting material proportioning program.

No.	CG	FA	Cement	Cement Ratio	Water-Cement Ratio	Water-Reducer
**S-1**	5	1	4	10:0	0.40	1.0%
**S-2**	5	1	4	9:1	0.40	1.0%
**S-3**	5	1	4	8:2	0.40	1.0%
**S-4**	5	1	4	7:3	0.40	1.0%
**S-5**	5	1	4	6:4	0.40	1.0%
**S-6**	5	1	4	5:5	0.40	1.0%
**S-7**	5	1	4	4:6	0.40	1.0%
**S-8**	5	1	4	3:7	0.40	1.0%
**S-9**	5	1	4	2:8	0.40	1.0%
**S-10**	5	1	4	1:9	0.40	1.0%

**Table 5:** EDS analyses of different SAC contents’ elements in weight percentages (unit: %).

Content	Position	O	Al	Si	C	Fe	Ca	K	Mg	Na
**0%**	Point-1	34.66	-	45.64	15.44	0.82	-	-	-	-
	Point-2	36.03	13.19	25.44	11.83	6.18	1.37	2.35	1.69	1.22
	Point-3	40.62	8.46	17.08	13.65	1.32	14.52	2.58	1.41	0.35
**40%**	Point-1	42.17	-	44.61	11.41	-	1.81	-	-	-
	Point-2	46.87	14.97	17.63	12.16	0.58	2.76	4.20	0.33	0.50
	Point-3	37.28	13.73	14.52	15.37	1.11	15.47	1.51	-	1.02
**90%**	Point-1	23.72	6.06	28.51	27.32	2.29	11.43	0.67	-	-
	Point-2	57.51	-	-	17.05	1.25	23.35	0.85	-	-
	Point-3	50.70	20.74	-	11.47	-	16.14	0.94	-	-

**Table 6:** EDS analysis for different fiber element weight percentages (unit: %).

Fiber	Position	O	Al	Si	C	Fe	Ca	K	Mg	Na
**BF**	Point-1	30.19	6.79	7.37	11.72	2.52	13.71	3.41	0.64	0.25
	Point-2	5.05	2.56	2.63	5.15	61.33	6.80	0.67	0.45	-
	Point-3	40.91	10.56	9.73	14.77	0.95	14.98	4.34	2.00	0.22
**GF**	Point-1	19.94	6.92	10.72	13.19	0.71	10.18	0.97	1.65	—
	Point-2	15.57	14.15	16.53	6.68	1.03	1.55	0.46	3.39	0.45
	Point-3	24.38	0.45	34.6	10.33	-	1.66	-	-	-
**PVA**	Point-1	41.51	5.60	6.48	26.06	0.38	12.00	3.34	0.67	0.13
	Point-2	29.74	14.34	15.88	6.24	4.40	21.58	2.57	4.13	0.23
	Point-3	51.52	12.53	14.62	12.03	0.63	7.04	0.74	0.63	0.26
**SF**	Point-1	36.76	5.61	4.25	14.24	0.38	13.81	3.97	0.47	-
	Point-2	45.50	7.69	19.53	12.00	-	1.85	-	0.32	-
	Point-3	26.65	3.04	19.44	19.84	0.54	3.15	0.75	0.56	-

## Data Availability

The original contributions presented in this study are included in the article. Further inquiries can be directed to the corresponding authors.
